# Staying the course

**DOI:** 10.1016/j.patter.2023.100690

**Published:** 2023-02-10

**Authors:** Andrew L. Hufton

**Affiliations:** Editor-in-Chief, *Patterns*

As our faithful readers will have already read in our January editorial, I have recently joined Cell Press and taken on the editor-in-chief role for *Patterns*. I look forward to building on the solid foundation laid by Sarah Callaghan, the journal’s founding editor-in-chief, and all of the other editors, authors, and reviewers who have contributed so much to growing and developing this new and innovative open access journal. Special thanks are due as well to Steve Cranford, who served as interim editor-in-chief during this transition.

The journal so far has been notable for its broad interdisciplinary scope, bringing together data science and data-heavy research from a wide range of fields. Rigorous, technical works are complemented with timely and broadly accessible opinions, reviews, and perspectives (explore all our article types). With artificial intelligence, data science, and the modern data deluge transforming so many research fields, I am committed to continuing and further expanding on this course. I want the journal to be as welcoming to data scientists as to philosophers and science policy scholars. I want the journal to be a bridge between the bleeding edge of technical data science and mainstream discussions about the myriad ways that data affect our everyday lives.

I also intend to continue the journal’s high standards for promoting FAIR, open, and reproducible science. The FAIRness and openness of data and computer code associated with submissions to the journal will be evaluated as an integral part of our editorial selection process. I encourage our authors to think not just about satisfying minimum sharing requirements but about how *best* to share their data and code. In general, we will expect data and code to be accessible to peer reviewers during their assessments. For content submitted under our descriptor article format, we will be particularly strict in enforcing these standards, since broad reuse value is our main criteria for these papers.

For works where restrictions on data or code sharing are needed, for example to protect privacy or ensure ethical or equitable use, we will work flexibly with our authors, respecting their limitations while seeking to develop peer-review processes that will meet the journal’s standards. We ask authors to describe and justify in their papers any limitations or special access conditions that may apply to their data or code. We strongly invite submissions from groups who have developed innovative sharing or access solutions for these kinds of challenging datasets.

The journal will also seek to ensure that our publications uphold the highest standards of research ethics, while being an unrelenting advocate for the responsible use of data in our wider society. As data, artificial intelligence, and other data science methods flood into all aspects of modern life, it is crucial that these innovations support global efforts to improve equity, protect human rights, and sustainably manage our planet.

For readers of the journal, these goals should feel familiar, reflecting the standard of excellence already evident in our past publications. *Patterns* is still a young journal, so we need your support to continue to achieve these aims. Please help us by submitting your best, data-rich research to the journal, by sending us ideas for new topics we should be covering, and by upholding the goals outlined above when reviewing for us.

